# Efficacy of bronchoscopic intratumoral injection of endostar and cisplatin in lung squamous cell carcinoma patients underwent conventional chemoradiotherapy

**DOI:** 10.1515/med-2023-0640

**Published:** 2023-04-03

**Authors:** Yanzhen Ji, Shuli Luan, Xiaoping Yang, Bin Yin, Xiaojie Jin, Haiyan Wang, Wenqing Jiang

**Affiliations:** Otorhinolaryngological Department, Hiser Medical Center of Qingdao, Qingdao 266033, Shandong, China; Department of Geriatrics, Hiser Medical Center of Qingdao, Qingdao 266033, Shandong, China; Pneumology Department, Hiser Medical Center of Qingdao, Qingdao 266033, Shandong, China; Pneumology Department, Hiser Medical Center of Qingdao, No. 4 Renmin Road, Qingdao 266033, Shandong, China

**Keywords:** lung squamous cell carcinoma, bronchoscopy, endostar, cisplatin, efficacy, adverse reactions

## Abstract

Bronchoscopy has been widely used for the therapy of lung cancer. This study aimed to evaluate the therapeutic efficacy and adverse reactions of bronchoscopic intratumoral injection of endostar and cisplatin in patients with lung squamous cell carcinoma (LSCC). A total of 40 LSCC patients who underwent conventional chemoradiotherapy were included in this study, and 20 of them received a bronchoscopic injection of endostar and cisplatin as an additive therapeutic modality (treatment group). The clinical response rate, progression-free survival (PFS), and adverse reactions of the patients were compared and analyzed. The treatment group had better short- and long-term therapeutic efficacy compared to the control group, but no significant differences were observed between the two therapeutic regimens in adverse reactions. Elderly and advanced LSCC patients had worse therapeutic efficacy and a high probability of adverse reactions after the therapy. Collectively, our analysis data demonstrated that the bronchoscopic intratumoral injection of endostar and cisplatin had improved therapeutic efficacy, and the cardiovascular adverse reactions were within the controllable range in the treatment of LSCC in clinical practices.

## 1 Introduction

Lung cancer, as a kind of frequent malignant tumor, accounts for the largest number of cancer-related deaths [1]. Non-small cell lung cancer (NSCLC) is the most common type of lung cancer, and lung squamous cell cancer (LSCC) accounts for about 30% of NSCLC [2]. Surgical treatment is preferred for those lung cancer patients who can still undergo surgery, but most of the patients have already reached advanced stages when they are diagnosed [3]. As a result, there are about 70% of patients lose the chance to undergo surgical treatment [4]. Therefore, systemic chemotherapy or transarterial chemotherapy combined with radiotherapy has become the major method for the treatment of lung cancer [5]. However, compared to small cell lung cancer (SCLC), NSCLC is not sensitive to chemotherapy, especially for LSCC [6]. Thus, specific and effective therapeutic approaches are necessary for the treatment of LSCC.

Bronchoscopes are suitable for observing pulmonary lobes, segment and subsegment bronchial lesions, biopsy sampling, bacteriology, and cytology examinations [7]. The bronchoscope is attached to a biopsy sampling structure, which helps detect early lesions and carry out internal surgery, such as polyp removal [8]. It is considered a good precision instrument for the research of bronchial and lung diseases. With the development of bronchoscopy in recent years, endoscopic interventional therapy plays an increasingly important role in the treatment of pulmonary diseases, especially lung cancer [9]. The intratumoral injection of anti-cancer drugs with the guidance of a bronchoscope is characterized by a simple operation and a high success rate, making it an efficient therapeutic method for cancer treatment [10]. However, the efficacy and safety evaluations of this method still need to be evaluated to provide more clinical evidence for its application.

Cisplatin is widely used as an anti-tumor drug in various malignant tumors, especially in NSCLC. It can bind to DNA, leading to the destruction of DNA function and inhibition of cell mitosis [11]. Recent studies have reported the considerable therapeutic efficacy of endobronchial intratumoral chemotherapy using cisplatin in lung cancer [12]. Next to chemotherapy, anti-angiogenic therapy also received increasing attention. The combinations of chemotherapy and anti-angiogenic therapy have presented to benefit patients with malignant tumors [13]. Angiogenesis plays a critical and promoting role in the progression of malignancies [14]. Anti-angiogenic agents, such as endostar (rh-endostatin), bevacizumab (anti-vascular endothelial growth factor A), and ramucirumab (anti-vascular endothelial growth factor R), have been applied for anti-tumor therapies, especially for tumors with advanced clinical stages [15]. It has been reported that the combination of endostar and chemotherapy significantly improves the clinical outcomes of cancer patients [16,17].

Our previous study found that the intratumoral injection of cisplatin and endostar with the guidance of a bronchoscope is an effective and safe adjuvant method to alleviate malignant central airway obstruction [18]. This study collected and analyzed the clinical data of LSCC patients who underwent conventional chemoradiotherapy combined with bronchoscopic intratumoral injection of endostar and cisplatin. The short- and long-term therapeutic efficacy and application safety were evaluated, and the clinical characteristics of patients that might affect efficacy and safety were further assessed. The analysis results of this study are expected to provide clinical evidence and data for the use of bronchoscopy in LSCC treatment.

## 2 Materials and methods

### 2.1 Study population

This study retrospectively analyzed 40 patients with LSCC who were admitted to Hiser Medical Center of Qingdao from December 2016 to June 2017. Twenty-eight males and 12 females were included, and the average age of the patients was 70.8 ± 1.8 years. According to tumor, node, metastasis (TNM) staging criteria, there were four cases of stage IIIB and 36 cases of stage IV. The inclusion criteria were as follows: (1) age of 18–80 years; (2) pathologically and/or cytologically confirmed as LSCC; (3) unresectable LSCC with central intraluminal growth; (4) expected survival time was more than 3 months; and (5) patients can understand the content of the study and had signed the informed consent. Following were exclusion criteria: (1) age >80 years or <18 years; (2) tracheal or carinal tumor obstructing more than 3/4 of the lumen; (3) external pressure airway stenosis; (4) severe arrhythmia, acute myocardial ischemia, and uncontrolled hypertensive crisis; (5) coagulation dysfunction; (6) severe organ dysfunction; (7) allergy to anesthetics; (8) pregnant and lactating women; and (9) intolerance to bronchoscopy and severe reaction to chemotherapy. All patients were considered unsuitable for surgical resection after multidisciplinary consultation and grouped into control group (*n* = 20) and treatment group (*n* = 20) based on the therapy received. Patients in the control group received conventional chemoradiotherapy, and patients in the treatment group received conventional chemoradiotherapy combined with bronchoscopic intratumoral injection of endostar and cisplatin. Each patient signed informed an consent form, and the study protocols were approved by the Ethics Committee of the Hiser Medical Center of Qingdao (#0015873).

### 2.2 Treatment regimens

For the control group, patients received conventional chemoradiotherapy, specifically systemic intravenous docetaxel combined with cisplatin or gemcitabine combined with cisplatin chemotherapy combined with radiotherapy. Each therapy cycle contained 21 days, and two consecutive cycles of chemotherapy were performed. Before the first cycle and after the second cycle, electronic bronchoscopy was used for tumor removal and biopsy.

For the treatment group, in addition to the conventional chemoradiotherapy, an electronic bronchoscope (Olympus BR-1T260) was used for the therapy. According to the routine operation of bronchoscopy, 2% lidocaine spray was used to achieve painless sedation before the operation, and a continuous intravenous infusion of propofol remifentanil was used during the operation. Cisplatin (20 mg) was dissolved in normal saline to 4 mL, and 15 mg endostar was also dissolved to 4 mL using normal saline. Under conventional chemoradiotherapy, endostar and cisplatin were locally injected with bronchoscopy on the third day (D3) and tenth day (D10) of each chemotherapy cycle. Routine bronchoscopy was performed, and bronchoscopic ablation was performed. After that, endostar and cisplatin were injected into the center and periphery of the residual tumors with four to six injection points. The penetration depth was 3–4 mm, and cisplatin and endostar with a volume of 0.5 mL were injected alternately each time. Two consecutive cycles of chemotherapy were applied.

The above two groups of treatment regimens were evaluated after two cycles of chemotherapy. At any time during the study, if there was objective evidence of tumor progression or serious adverse events, the treatment would be terminated.

### 2.3 Evaluation of therapeutic efficacy

After two cycles of therapy, therapeutic efficacy was evaluated and compared. The short-term therapeutic efficacy was defined as follows: complete remission (CR), the complete elimination of tumors in the airway, and lasted for 1 month; significant remission (SR), where the minimal diameter of tracheobronchial stenosis increased by more than 30% after the treatment, lasting for 1 month; minor remission (MR), where the minimal diameter of tracheobronchial stenosis increased by less than 30% after the treatment, lasting for 1 month; and no response (NR), where the minimal diameter of tracheobronchial stenosis decreased by more than 30%. The clinical remission rate (CRR) was calculated as (CR + SR)/total cases × 100%, and the clinical beneficial rate (CBR) was calculated as (CR + SR + MR)/total cases × 100%.

For long-term therapeutic efficacy, the progression-free survival (PFS) of each patient was evaluated. All the patients were followed up to record the progression of the disease, and PFS was defined as the time between initial chemotherapy and disease progression or death.

### 2.4 Adverse reaction data collection

Adverse reaction evaluation was performed in accordance with the Common Terminology Criteria for Adverse Events (CTCAE) by the National Cancer Institute of the USA [19]. Myelosuppression, liver dysfunction, and gastrointestinal reactions presented after the treatment were analyzed. To evaluate myelosuppression, cases of leucopenia, neutropenia, haemoglobinia, and thrombocytopenia were recorded. The concentrations of glutamic-pyruvic transaminase (ALT) and aspartate transaminase (AST) were measured to evaluate liver function. Gastrointestinal reactions mainly included nausea and emesis. In addition, the cardiovascular adverse reactions were evaluated using an electrocardiogram. Electrocardiographic abnormalities included changes in the ST segment and T wave (ST-T changes). Blood pressure fluctuation was defined as that which increased or decreased by ≥20 mmHg compared with that before treatment (excluding fluctuation within the normal range).

### 2.5 Statistical analysis

SPSS statistical software 26.0 was used to perform statistical analyses in this study. The data were expressed as frequency and/or percentage and analyzed using the Chi-square test or F test. The PFS of patients was analyzed using the Kaplan–Meier method, and the distributions between the curves were compared using the log-rank test. A difference with *P* < 0.05 indicated statistically significant.

## 3 Results

### 3.1 Baseline characteristics of the LSCC patients

The baseline features of the patients were listed in Table 1. Patients in the treatment group were 70.6 ± 1.2 years old and contained 15 males and five females. The control group was age- and gender-matched with the treatment group (both *P* > 0.05), which contained 13 males and seven females and aged 70.9 ± 2.2 years. There were 15 former smokers in the treatment group and 14 in the control group. Both groups had two cases with IIIB-stage tumors and 18 cases with IV-stage tumors.

**Table 1:** Baseline characteristics of the study population

Characteristics	Total no.	Control group (*n* = 20)	Treatment group (*n* = 20)	*P* value
Age (years)				0.752
≤70	19	10	9	
>70	21	10	11	
Gender				0.490
Female	12	7	5	
Male	28	13	15	
Smoking				0.723
Never	11	6	5	
Ever	29	14	15	
TNM stage				1.000
IIIB	4	2	2	
IV	36	18	18	

### 3.2 Short- and long-term therapeutic efficacy between the two groups

After the two cycles of therapy, short-term therapeutic efficacy was evaluated. There were six SR cases, 10 MR cases, and four NR cases in the control group, and the CRR and CBR were 30 and 80%, respectively. No CR patients were observed in this group. For the treatment group, all the patients had varying degrees of remission, and there were four CR cases, 14 SR cases, and two MR cases with CRR and CBR of 90 and 100%, respectively. Compared to the control group, the patients in the treatment group showed significantly better efficacy (*P* < 0.001, Table 2).

**Table 2:** Comparison of short-term therapeutic efficacy between the two groups

Groups	Total no.	CR	SR	MR	NR	CCR(%)	CBR(%)	*P* value
Control group	20	0	6	10	4	30	80	0.001
Treatment group	20	4	14	2	0	90	100	

CCR, (CR + SR)/total cases × 100%; CBR, (CR + SR + MR)/total cases × 100%.

**Figure 1:**
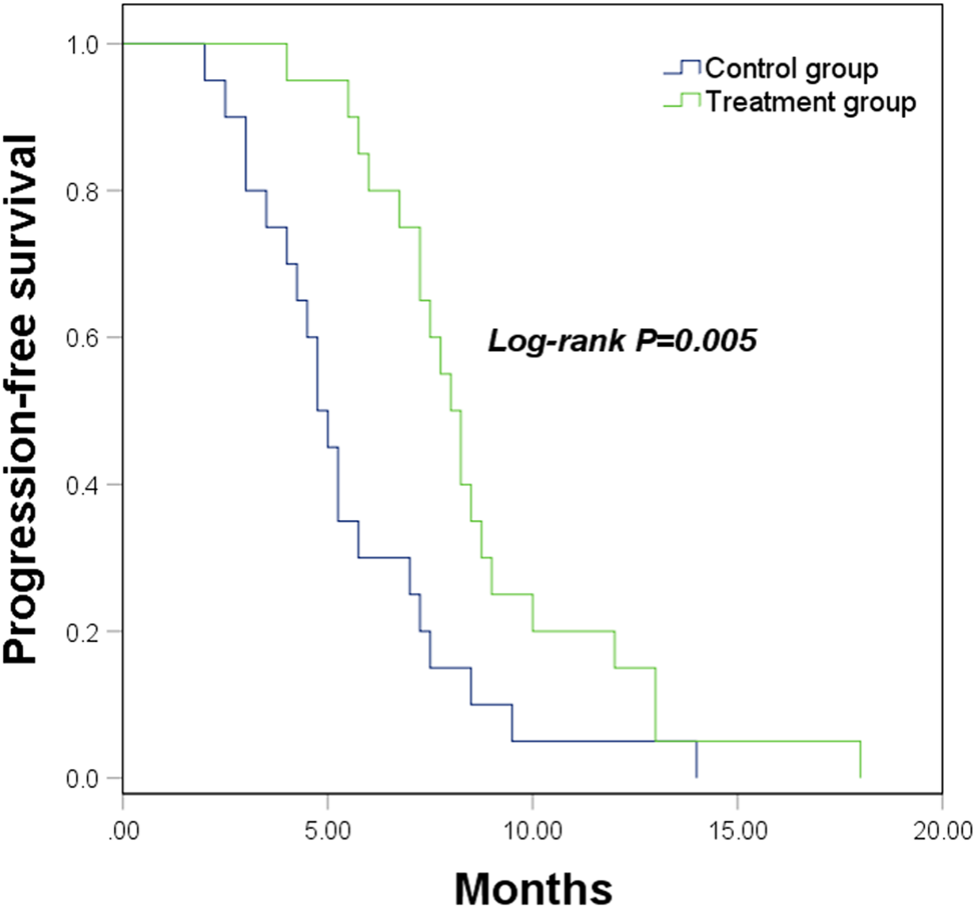
PFS of LSCC patients underwent different therapeutic methods. log-rank *P* = 0.005.

This study followed up on the disease progression of patients to evaluate long-term efficacy in the two groups. The follow-up time was 5.6 ± 2.8 months (range: 2–14 months) for the control group and 8.7 ± 3.2 (range: 4–18 months) for the treatment group. The median PFS of the control group was 4.8 months, the median PFS of the treatment group was 8.0 months, and the PFS of the treatment group was markedly better than that of the control group (log-rank *P* = 0.005, Figure 1).

### 3.3 Adverse reactions after the treatment of the two groups

Patients in the control group developed 12 leucopenia, 11 neutropenia, nine haemoglobinia, three thrombocytopenia, four ALT increase, three AST increase, six nausea, and five emesis cases. No cardiovascular adverse reaction was observed in this group. In the treatment group, there were 13 leucopenia, 10 neutropenia, nine haemoglobinia, one thrombocytopenia, three ALT increase, three AST increase, four nausea and four emesis cases, and one case with ST-T change and two cases with blood pressure fluctuation. According to the CTCAE, the adverse reactions (in addition to the cardiovascular adverse reactions) are graded into I–II and III–IV grades (Table 3), and no significant differences were observed in these reactions between the two groups (all *P* > 0.05).

**Table 3:** Comparison of adverse reactions between the two groups after therapy

Parameters	Control group (*n* = 20)	Treatment group (*n* = 20)	*P* value
Leucopenia	12	13	0.801
I–II	6	8	
III–IV	6	5	
Neutropenia	11	10	0.907
I–II	7	7	
III–IV	4	3	
Haemoglobinia	9	9	0.819
I–II	7	8	
III–IV	2	1	
Thrombocytopenia	3	1	0.292
I–II	3	1	
III–IV	0	0	
Increased ALT	4	3	0.677
I–II	4	3	
III–IV	0	0	
Increased AST	3	3	1.000
I–II	3	3	
III–IV	0	0	
Nausea	6	4	0.465
I–II	6	4	
III–IV	0	0	
Emesis	5	4	0.705
I–II	5	4	
III–IV	0	0	
ST-T changes	0	1	0.311
Blood pressure fluctuation	0	2	0.147

### 3.4 Relationship between therapeutic efficacy and clinical characteristics in the treatment group

To find the factors that might affect the therapeutic efficacy of the treatment group, the clinical responses of patients were compared between patients with different clinical features. As shown in Table 4, patients aged ≤70 years had more cases with CR and SR conditions compared with those aged >70 years (*P* = 0.030). All of the two IIIB-stage patients showed CR and were significantly different in clinical responses from the patients with IV tumors (*P* = 0.012).

**Table 4:** Association of short-term efficacy with clinical data in the treatment group

Variables	CR	SR	MR	*P* value
Age (years)				0.030*
≤70	4	5	0	
>70	0	9	2	
Gender				0.683
Female	1	4	0	
Male	3	10	2	
Smoking				0.683
Never	1	3	1	
Ever	3	11	1	
TNM stage				0.012*
IIIB	2	0	0	
IV	2	14	2	

*P < 0.05.

**Figure 2:**
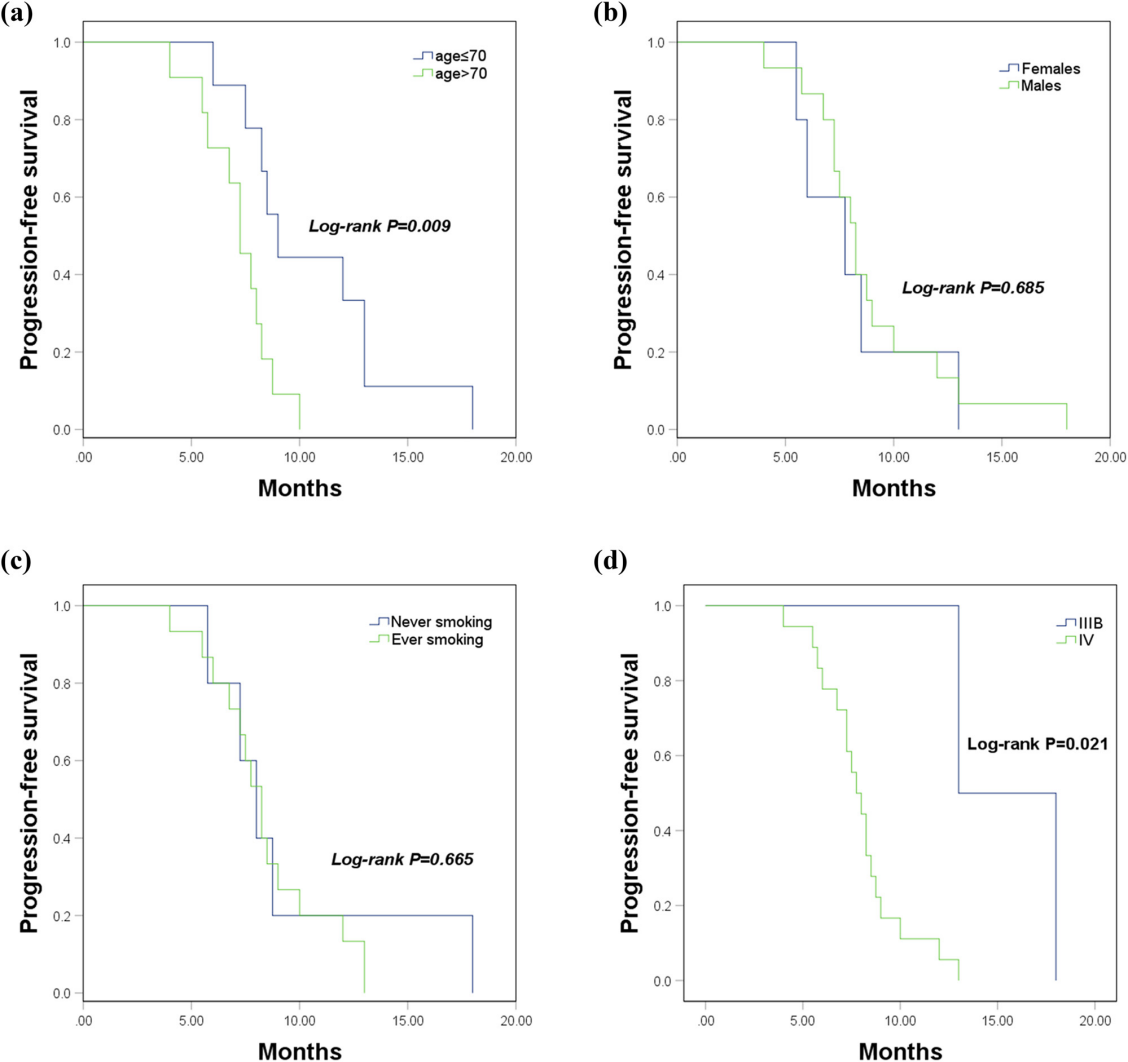
Association of clinical data with the PFS of LSCC patients in the treatment group. (a) PFS of patients with different ages (log-rank *P* = 0.009). (b) PFS of patients with different gender (log-rank *P* = 0.685). (c) PFS of patients with different smoking history (log-rank *P* = 0.665). (d). PFS of patients with different TNM stages (log-rank *P* = 0.021).

In addition, the PFS curves were further plotted in patients with different clinical data, the PFS in the population >70 years old was poorer than that in the patients who were ≤70 years old (log-rank *P* = 0.009, Figure 2a). No differences were found in the PFS between patients with different genders or smoking history (log-rank *P* > 0.05, Figure 2b and c). Patients at IIIB stage had a better PFS compared to those at the IV stage (log-rank *P* = 0.021, Figure 2d).

### 3.5 Reverse reactions in patients with different clinical features in the treatment group

The patients in the treatment group were divided into multiple subgroups based on their clinical features, and the adverse reactions were compared between the subgroups. The comparison results were listed in Table 5, which showed that patients who were >70 years old had significantly more leucopenia and neutropenia cases (both *P* < 0.05), and that more leucopenia cases were observed in IV-stage patients compared to IIIB-stage patients (*P* < 0.05). However, no relationship between other clinical features, including smoking history and gender, with the adverse reactions was found in this analysis (all *P* > 0.05).

**Table 5:** Association of reverse reactions with clinical data in the treatment group

Parameters	Age (years)		Gender		Smoking		TNM stage	
	≤70	>70	Female	Male	Never	Ever	IIIB	IV
Leucopenia	3	10	3	10	4	9	0	13
*P* value	0.007*		0.787		0.417		0.042*	
Neutropenia	2	8	2	8	3	7	1	9
*P* value	0.025*		0.606		0.606		1.000	
Haemoglobinia	4	5	2	7	2	7	0	9
*P* value	0.964		0.795		0.795		0.178	
Thrombocytopenia	0	1	0	1	0	1	0	1
*P* value	0.353		0.554		0.554		0.732	
Increased ALT	1	2	1	2	0	3	0	3
*P* value	0.660		0.718		0.278		0.531	
Increased AST	0	3	1	2	1	2	1	2
*P* value	0.089		0.718		0.718		0.144	
Nausea	1	3	1	3	1	3	0	4
*P* value	0.369		1.000		1.000		0.456	
Emesis	1	3	2	2	1	3	1	3
*P* value	0.369		0.197		1.000		0.264	
ST-T changes	0	1	0	1	0	1	0	1
*P* value	0.353		0.554		0.554		0.732	
Blood pressure fluctuation	0	2	1	1	0	2	0	2
*P* value	0.178		0.389		0.389		0.619	

*P < 0.05.

## 4 Discussion

In malignant tumors, intratumoral injection of chemotherapeutic drugs combined with cryotherapy and hyperthermia is a good cooperative therapeutic strategy for disease treatment [20]. A study by Weill et al. reported the data from 12 lung cancer patients (six LSCC cases and six lung adenocarcinoma cases) who underwent intratumoral injection of recombinant human p53 adenovirus under bronchoscopy, which found that the airway obstruction of 50% patients was relieved, and three cases in the 12 patients were partially relieved [21]. Local injection through the bronchoscope can obviously increase drug concentration in tumors and is characterized by a small drug dosage and obviously reduced adverse reactions [22]. Due to the high local drug concentration in tumors and long drug action time, tumors shrink rapidly, and thereby the airway obstruction and ventilation were significantly relieved, improving the quality of life and prolonging survival in lung cancer patients. Currently, the commonly used drugs for intracavitary injection include chemotherapy drugs (cisplatin, mitomycin, and epirubicin), absolute ethanol, interleukin-2 (IL-2), and gene-based drugs (recombinant human p53 adenovirus injection), but the drugs above are still in the exploratory stage [23]. This study aimed to investigate the combined injection of endostar and cisplatin with the hope to provide novel ideas for the therapy of LSCC.

Cisplatin is a kind of non-specific cytotoxic drug and plays a pivotal role in the treatment of lung cancer [24]. As we all know, cisplatin is a dose-dependent drug, meaning that the higher the concentration, the greater the killing effect on tumor cells. However, high doses of cisplatin cause serious adverse reactions, leading to its limited clinical application [25]. Previous reports have confirmed that after the body cavity injection of cisplatin, the peak concentration and concentration-time curve area in the cavity were 20 times and 12 times that in the plasma, respectively, which improved the effect of chemotherapy [26]. At the same time, about 60% of the intracavitary drugs can be absorbed into the systemic circulation and re-enter the tumor tissue to kill tumor cells [27]. Thus, there are many basic and clinical trials that have used an intracavitary infusion of cisplatin to treat malignant pleural effusion and pericardial effusion, and good results have been achieved [28,29]. Therefore, local tumor injection of cisplatin should also be able to achieve the purpose of efficient killing of tumor cells and effectively reduce the toxic and side effects of systemic medication. This corollary has been confirmed in several studies, which provided evidence for intratumoral injection of cisplatin [30,31].

Endostar with its full name of recombinant human endostatin injection is the first endostatin-based new drug for lung cancer. It has a significant biological function, including tumor neovascularization inhibition and promotion of cancer cell apoptosis. As our innovation, this study added endostar to the local injection of cisplatin. There is no indication of local use of endostar in the instructions till now, and the relevant study to observe the dosage of local use of endostar was also rare. In order to provide more ideas for the treatment of LSCC, we chose the intratumoral injection method of cisplatin combined with endostar. Through our observation, we found that the intratumoral injection of cisplatin and endostar under bronchoscope, especially combined with the freezing and hyperthermia technology, significantly eliminated the tumor in time, controlled the tumor growth, maintained the patency of the tracheal and bronchial lumens, prolonged the time of tracheal restenosis, and had no obvious complications, which is a rapid, effective, and safe interventional treatment method under bronchoscope. Compared to the LSCC patients who underwent conventional chemoradiotherapy, intratumoral injection of endostar and cisplatin had better short- and long-term therapeutic efficacy, which was evidenced by the markedly increased CRR and CBR, and improved PFS in the treatment group.

Endostar has a significant anti-tumor angiogenesis effect and can significantly reduce the expression of vascular endothelial growth factor (VEGF), which has a greater effect on inhibiting tumor infiltration and metastasis [32]. VEGF is a multifunctional cytokine that interacts directly with vascular endothelial cells, exerting a strong vasoactive effect, increasing the permeability of microvessels, and promoting the infiltration and metastasis of tumor cells [15]. Previous studies showed that endostar can directly inhibit the proliferation, migration, and differentiation, promote the apoptosis of vascular endothelial cells, and antagonize the role of VEGF in promoting angiogenesis and increasing vascular permeability [33]. It has the advantages of a broad anti-tumor spectrum, low toxicity, and no drug resistance and plays a synergistic role when combined with cisplatin [34].

Our previous study has reported the improved therapeutic efficacy of bronchoscopic intratumoral injection of endostar and cisplatin in the treatment of malignant central airway obstruction [18]. In the present study, we further assessed the adverse reactions to evaluate the safety of this therapeutic method. In the treatment group of this study, myelosuppression was the major adverse reaction, such as leucopenia, neutropenia, and haemoglobinia. In addition, one ST-T change case and two blood pressure fluctuation patients were observed after the treatment. Considering the causes of the adverse reactions, the myelosuppression, liver dysfunction, and gastrointestinal reactions might be caused by conventional chemotherapy, which is consistent with previous reports [35]. The cardiovascular adverse reactions only showed up in the treatment group, which were considered to be related to the use of endostar, but most of them disappeared within 1–2 weeks after the therapy without any hindrance to the subsequent therapy. Compared with the control group, in which patients only received conventional chemoradiotherapy, the treatment group had no significant differences in adverse reactions, indicating the acceptable safety of bronchoscopic intratumoral injection of endostar and cisplatin for the treatment of LSCC.

To discover the factors that might affect the therapeutic efficacy and adverse reactions of the intratumoral injection method, the patients in the treatment group were further divided into multiple subgroups based on their clinical characteristics. The age and TNM stage were found to be significantly associated with efficacy and the onset of adverse reactions. Compared with younger or early-stage tumor patients, older patients or advanced tumor patients had worse therapeutic efficacy and a higher probability of adverse reactions after endostar and cisplatin injection. These findings are in accordance with the conclusions from previous studies, which reported that age and TNM stage are two important factors related to clinical outcomes in cancer patients [36]. Thus, the monitoring and prevention of adverse reactions should be strengthened in the elderly and advanced LSCC patients after the treatment.

Some limitations were included in this study, and the first and most important was the small sample size. The data from only 20 patients who received a bronchoscopic intratumoral injection of endostar and cisplatin were collected and analyzed. More evidence is necessary for larger cohorts. Second, this study failed to analyze the data from patients who received therapy with different administration methods. Adverse reactions might be reduced by different administration methods. Therefore, further studies are warranted using larger study populations and a variety of dosing methods.

To sum up, this study provides clinical evidence for the application of bronchoscopic intratumoral injection of endostar and cisplatin as an adjuvant therapeutic strategy for the treatment of LSCC. The analysis of the data from 20 LSCC patients indicated that endostar and cisplatin intratumoral injection had better short- and long-term therapeutic efficacy and had no significant additional adverse reactions compared to conventional chemoradiotherapy. Endoscopic injection of endostar may have some cardiovascular adverse reactions, but most of them are transient and reversible and have no obvious effect on the subsequent treatment. In addition, in the clinical practices of endoscopic injection of endostar and cisplatin, it is necessary to consider the age and clinical stage of patients, and dynamic monitoring of the electrocardiogram and clinical symptoms are also needed. However, the small sample size may limit the accuracy of the analysis results. A larger study population may provide some interesting information and indications.
